# The knowledge, attitudes, and behaviours of pregnant women regarding seafood consumption during the antenatal period: a qualitative study

**DOI:** 10.1186/s12884-023-06149-5

**Published:** 2024-01-24

**Authors:** Danielle Shine, Heshani Siriwardana, Michelle Minehan, Monica Yuri Takito, Rati Jani, Catherine R. Knight-Agarwal

**Affiliations:** 1 Department of Nutrition and Dietetics, The University of Canberra, Locked Bag 1, ACT, Bruce, Bruce, Australia; 2 Department of Human Movement, The University of São Paulo, São Paulo, Brazil

**Keywords:** Diet quality, Nutrition, Pregnancy, Health promotion, Fish and seafood, Omega 3 fatty acids, Fetal development

## Abstract

**Background:**

Maternal nutrition impacts fetal growth and development. The Food Standards Australia New Zealand (FSANZ) guidelines recommend pregnant women consume 2–3 servings (224–336 g) of fish/seafood per week to support intake of long chain omega 3 fatty acids, given adequate consumption supports numerous health benefits including reduced risk of preterm and early preterm birth. Evidence indicates that pregnant women purposely lower their fish/seafood intake, largely due to fears of methylmercury exposure. The aim of this study was to explore pregnant women’s knowledge, attitudes, and behaviours regarding their fish/seafood consumption during the antenatal period.

**Methods:**

Semi-structured interviews were conducted between October 2018 and December 2020 among a purposive sample of 12 pregnant women from the Australian Capital Territory (ACT). The interviews were recorded, transcribed verbatim, and analysed using an interpretative phenomenological approach. Themes were developed on the women’s lived experience related to fish/seafood knowledge, attitudes, and consumption behaviour.

**Results:**

The most prominent finding was widespread non-adherence to fish/seafood consumption guidelines. This was largely owing to a lack of proactive health promotion related to the health benefits of fish/seafood throughout pregnancy, including the health promoting roles of long chain omega 3 fatty acids for fetal growth and development. Three themes were identified: nutrition knowledge; sources of health promotion; and barriers and enablers to fish/seafood consumption.

**Conclusions:**

To support adequate maternal consumption of fish/seafood throughout pregnancy, emphasis should be placed on the benefits of consuming this food group regularly. Additionally, pregnant women should receive education about the health promoting role of long chain omega 3 fatty acids. Dietitians are well placed to provide this information.

## Background

Maternal nutrition is a major contributor to human health; it plays a significant role in fetal development and may have long-term consequences to health in adult life. Fish/seafood is an excellent source of the long chain omega 3 fatty acids eicosapentaenoic acid (EPA) and docosahexaenoic acid (DHA) in addition to other key nutrients, such as vitamin D, vitamin B_12_, iodine, and an array of highly bioavailable proteins [1]. In Australia, Clinical Practice Guidelines support regular maternal intake of fish/seafood during pregnancy, as it is likely to have a range of health benefits for women and their children but, in turn, emphasise that fish should be low in mercury [2]. The most common naturally occurring sources of EPA/DHA are oily fish varieties including salmon, mackerel, and sardines. A 2018 Cochrane review conducted by Middleton et al. [3] synthesised evidence from 70 randomised controlled trials of EPA/DHA supplementation during pregnancy. The authors reported that long chain omega 3 fatty acid intervention during pregnancy reduced the risk of preterm birth by 11% and early preterm birth (< 34 weeks’ gestation) by 42%. This is important, given preterm birth (< 37 weeks) has the highest burden in terms of mortality and morbidity of all pregnancy-related adverse outcomes. Worldwide, there are approximately 15 million preterm births per year, accounting for 85% of all perinatal complications. It is the leading cause of death in children aged < 5 years of age. Researchers concluded that for pregnant women to obtain the dose of long chain omega 3 fatty acids effective for reducing preterm birth they would need to eat at least two serves (~ 150 g per serve) of salmon, for example, per week or consume the equivalent in supplements or fortified foodstuffs [3]. This is in line with current advice from the United States (US) Environmental Protection Agency and the US Food and Drug Administration [4] that recommend pregnant women consume 224 to 336 g (8–12 oz) (2–3 servings) of most types of commercially available fish/seafood per week. The advice stems from evidence of the significant benefits to fetal growth and development. Additionally, FSANZ published similar guidance, at Table 1, that encompasses additional recommendations related to the safe consumption of predatory fish that, due to their larger size and longer lifespans, are more likely to contain higher levels of mercury [5].

**Table 1:** The size and number of portions of fish recommended for safe consumption in Australia, including during pregnancy [5]

Women who are pregnant or planning pregnancy 1 portion is 150 g^#^	Children (up to 6 years) 1 portion is 75 g^#^	Rest of the population 1 portion is 150 g^#^
1 portion per week of Orange Rough (Deep Sea Perch) or Catfish and no other fish that week ​		​​1 portion per week of Shark (Flake) or Billfish (Swordfish/Broadbill and Marlin) and no other fish that week
OR		
​1 portion per fortnight of Shark (Flake) or Billfish (Swordfish/Broadbill and Marlin) and no other fish that fortnight ​		

Nevertheless, there is evidence that pregnant women are not achieving recommended intakes of long chain omega 3 fatty acids. For example, an Australian cohort study [6] compared the dietary intake of women with national recommendations. The authors reported that pregnant women’s mean (including standard deviation fish consumption was 28.2 (37.4)g/day, and for women who had given birth within the last 12 months, 27.8 (26.6)g/day. These intakes were significantly lower compared to non-pregnant women who were reported to consume 33.0 (37.4) g/day (P = 0.01 and P < 0.001, respectively), in addition to being well below the latest targets supported by FSANZ [5].

Many women alter their consumption when pregnant for myriad reasons namely fear of exposure to methylmercury [7] as well as the risk of ingesting bacteria such as listeria monogenesis [8]. The most influential study concerning the possible dangers of eating fish/seafood during pregnancy came from the Faroe Islands where the level of methylmercury in maternal hair and cord blood was shown to be negatively associated with cognitive abilities of the offspring at age 7 years [9]. Although it was clearly stated that the methylmercury levels were associated with consumption of pilot whale (a sea mammal, not a fish), the subsequent assumptions were that fish/seafood in general was responsible for increased levels in the mother, and hence the fetus, and that methylmercury levels in pregnancy should be minimised [9]. Consequently, dietary advice for pregnant women has generally been that fish/seafood is an excellent source of many nutrients with an accompanying caveat to avoid varieties known to have particularly high levels of methylmercury [9]. From a psychosocial perspective, this latter message is the one that women have remembered with the general reaction having been to reduce their intake of all fish/seafood during pregnancy [10]. To impact this issue further, Australian qualitative research has reported that much of the dietary information available to pregnant women (including recommended fish/seafood intakes) via health professionals, friends, family, and social media tends to emphasise what not to eat rather than what to eat [11]. Other research has reported barriers for fish/seafood consumption during pregnancy, including the cost, availability, quality of produce and sensory aversions [12].

Several years have passed since an Australian-based qualitative study [12] was undertaken to explore pregnant women’s fish/seafood consumption. It is therefore important to revisit and update the literature related to this. The aim of our research was to qualitatively explore pregnant women’s knowledge, attitudes, and behaviours related to fish/seafood consumption.

## Methods

Qualitative research is often used to gain a better appreciation of an individual’s underlying behaviours and feelings. We consider reality to be socially constructed aiming to produce subjective findings via a process of inductive reasoning [13]. Rather than setting up a series of hypotheses, the research presented here has been guided by a semi-structured interview schedule that centers around experience. As a result, we deemed it most appropriate to employ Interpretative phenomenological analysis, as our qualitative approach, due to the topic under investigation being concerned with women’s’ lived experience of consuming fish/seafood during pregnancy and how they then make sense of that experience [14].

Purposive sampling requires that individuals are deliberately selected with a specific purpose in mind, predominantly to address the research aim and because they are rich sources of data in relation to this [13]. A university campus, situated in a metropolitan region of South-Eastern Australia, was chosen as the main site for recruitment, with study information circulated via the institution’s intranet and via online social media platforms. This campus includes two general practitioner (GP) clinics and a school of midwifery as part of the faculty of health. Women were recruited through the antenatal service they were attending. It is common for pregnant women in Australia to select a shared care arrangement between their GP and local public hospital. Alternatively, some women choose to receive their antenatal care through Midwifery or Obstetric-led clinics in the public hospital system or from a private practicing Obstetrician or Midwife. All the women in our study received their antenatal care via one of these care pathways. Flyers advertising the study were positioned around the university and at various health facilities within the ACT. In addition, details about the study were posted on the university’s health intranet.

Participants in our study were selected based on their personal characteristics (e.g., pregnant), their experience of a specific issue (e.g., pregnancy-related information) and their behaviour (e.g., fish/seafood consumption). Women met inclusion criteria if they were aged 18 years or older, were of gestation ≥ 12 weeks and resided in the ACT. Twelve women agreed to be interviewed. Each woman provided signed consent and was given a unique identifying number to ensure anonymity. A semi-structured interview guide was developed by the research team based upon a review of the published literature.

Interviewers (HS, CKA and MYT) attended interview training techniques together to ensure a standardised process was followed [14]. Interviews were conducted between October 2018 and December 2020. Participants elected a time and place for the interview that was convenient for them. Questions were kept deliberately open providing cues for participants to talk openly and without judgement. A faciliatory interview style was employed which included the use of verbal and non-verbal cues. Each interview was audio recorded and transcribed verbatim and then entered into a word processing document for analysis. The twelve transcripts were coded in detail to single out recurring patterns and quotes were given a unique qualifier to identify each participant. This process was undertaken iteratively in addition to ongoing discussion between all researchers to ensure rigour of findings and to determine the point at which data saturation was achieved. This process also served as an important means of triangulation and, as a result, the final set of superordinate themes was decided on [14].

## Results

Data analysis revealed three themes: Nutrition knowledge – what women know about eating fish/seafood in pregnancy; Sources of health promotion and advice about fish/seafood consumption, and appropriate alternatives, during pregnancy; Barriers and Enablers to pregnant women’s fish/seafood consumption.

### Theme 1: Nutrition knowledge – what women know about eating fish/seafood in pregnancy

Participants strongly believed that nutrition was important during pregnancy; most spoke about how they made an effort to eat healthily, given this directly impacted their developing fetus/baby:

*“When you’re pregnant, you’re growing a life inside of you, so even more so it’s important to know what you’re putting in your body.”* (P6).

For some, prevention of adverse birth outcomes was a motivator to eat health supportive foods throughout pregnancy:

*“Nutrition is important to me, because I don’t want to be put at risk for high blood pressure or gestational diabetes, gaining too much weight and all the other complications that come with not being healthy.”* (P4).

Despite the general view that antenatal nutrition is important, some women were not able to articulate what a healthy diet should ‘look like’ as stated by the following participant:

*“What kind of foods? I guess a balance … food from the pyramid thingy.”* (P1).

Participants were asked about the value of long chain omega 3 fatty acids during pregnancy. This rendered a wide spectrum of responses. From *“I have no idea (what they do)”* (P5), *“It’s good for your skin … that’s all I know.”* (P8), to *“Eating foods rich in omega 3’s will help with my babies brain development.”* (P10).

More specifically, when women were quizzed about their knowledge of pregnancy-related fish consumption guidelines many declared steering clear of ocean produce all together due to food safety worries as voiced by the following participants:

*“I avoid it (fish/seafood) during pregnancy because it comes with more risks.”* (P4) and *“My husband bought me a beautiful piece of swordfish which I didn’t eat the other day (due to my concern), I was disappointed about that…”* (P2).

Conversely, the following participant stated:

*“I didn’t really think there was a limit on how much (fish/seafood) you should consume … I thought it was more about the type like eating raw seafood.”* (P7).

Meanwhile, others voiced their motivation to regularly consume ocean fare during pregnancy acknowledging this food group to be an important part of a healthy diet:

*“I eat salmon and fatty fish … I know that it is good for my body in a lot of ways … it gives me a lot of nutrients.”* (P6).

Many women reported that they were taking a common pregnancy-specific multivitamin available through their local pharmacy. However, as a result, some believed it was then acceptable to omit long chain omega 3 fatty acid rich foods, such as fish, from their diet holding the misconception that: *“I know it (must) be in my multivitamin.”* (P4).

Overall, there was a lack of knowledge regarding appropriate dietary alternatives to fish/seafood for obtaining recommended amounts of long chain omega 3 fatty acids when pregnant. Only a small number of women were able to offer some feasible replacements:

*“…flaxseeds, I’m pretty sure … (they are an appropriate alternative).”* (P5).

### Theme 2: Sources of health promotion and advice about fish/seafood consumption, and appropriate alternatives, during pregnancy

Participants in our study spoke of getting little-to-no information about the importance of long chain omega 3 fatty acid intake during pregnancy from their antenatal care providers:

*“The only kind of diet advice I got from my doctor was specific things to avoid, and she didn’t mention seafood (as important).”* (P10).

Participants who had experienced a previous pregnancy recalled being given a general healthy eating *“…booklet”* at their first ever antenatal care appointment. Nevertheless, the following woman commented:

*“I can’t remember if they provided me advice about fish in my last pregnancy, but I know I haven’t been provided advice this pregnancy.”* (P4).

Likewise, another participant suggested that health professionals rely on women to seek out their own dietary information, especially:

*“…when it’s your second pregnancy, I think they kind of take for granted that you know (or know where to access new information from).”* (P6).

Others spoke about receiving links to general *“…websites”* and a *“…pregnancy lifestyle guide”* (P7), however, could not recall any information related to fish/seafood consumption, including how much fish was safe to eat.

Smartphone applications (apps) were utilized by some women for the receipt of nutrition advice:

*“I think (the one I used) is by an American company and it comes up with articles as well … You can go to the food safety (section), and it will tell you know what to eat or maybe eat only a small amount.”* (P8).

Along similar lines, others claimed that they had been forced to search the internet themselves for pregnancy-related nutrition information as the following participant lamented:

*“I googled … general sort of articles that say what fish to avoid, the main thing I probably looked up was whether I was supposed to eat canned tuna or not.”* (P7).

Another participant offered the following insight:

*“I’m very mindful about what information I get from online just because I feel like a lot of it is people telling you their opinions and thoughts and trying to pass it off as a fact.”* (P9).

Concerningly many participants shared information they had obtained about fish/seafood consumption that was either inaccurate or incorrect:

*“****…****well, I know you can’t eat the deep-sea fish because the increased level of mercury.”* (P6).

*“I would probably eat more tuna when I was before I was pregnant, but it has a higher risk of mercury, so I have stopped the tuna.”* (P11).

*“I’m not sure how safe it is to eat canned tuna, so I’ve had to cut that out.”* (P5).

For participants who ate little-to-no fish, knowledge about recommended fish/seafood intake during pregnancy was not a high priority:

***“****I don’t eat fish more than once a week so I am not thinking about it… it doesn’t matter what type of fish I eat if I’m only eat it once a week then, yeah, I feel like I should be fine.”* (P8).

The widespread lack of health promotion may explain why most participants did not adhere to evidence-based guidelines related to fish/seafood intake during pregnancy with only one participant appearing to consume the recommended 2–3 serves per week:

*“…fish is something which is very much part of my diet … I probably have fish at least twice a week sometimes three times a week.”* (P9).

### Theme 3: Barriers and Enablers to pregnant women’s fish/seafood consumption

There were a number of barriers to women consuming fish/seafood, particularly fish, during pregnancy. Searching for nutrition information inadvertently seemed to act as a barrier to safe and adequate fish/seafood consumption:

*“There is so much … it just overwhelms you.”* (P3).

Participants’ fear of methylmercury ingestion was a significant barrier to fish/seafood consumption. Despite not being asked about this neurotoxin specifically, the majority of participants cited it as something that they were wary of:

*“…most of the seafood that lives in the sea will have it (mercury).”* (P1).

*“I avoid it (fish/seafood) during pregnancy because it comes with more risks … there’s a lot of things regarding the buildup of mercury in certain fish.”* (P3).

For some women the cost associated with fish/seafood was a barrier to intake. For example, many women found fish/seafood to be *“…quite expensive”* (P1), while others admitted rarely purchasing their favourite fish throughout pregnancy, given it was *“…pricey.”* (P5).

An aversion to fish/seafood acted as a barrier for many:

*“…the smell just puts me off.”* (P4).

*“For a long time, when I was not feeling very well (during pregnancy) I didn’t really feel like eating it (fish/seafood).”* (P7).

*“I got food poisoned a few years ago and that was it … I stopped eating fish ever since.”* (P12).

Additionally, participants who lacked cooking skills also consumed less fish/seafood throughout their pregnancy:

*“…the reason that I don’t have it that often is that I’m not very good at cooking.”* (P10).

Overall, there were only a small number of participants who didn’t consume any fish/seafood. Personal preference was the main reason for abstinence:

*“I became a vegetarian when I was 13.”* (P4).

The main enablers to fish/seafood consumption were also identified. These included participants’ high motivation to eat well throughout pregnancy to not only support the health of themselves but their growing baby too:

*“…certainly, the third trimester I have been much more conscious about the quality of the food that I’m eating and the timeliness of it.”* (P2).

*“Before I was pregnant, I would still eat fish, but I could go a few weeks without eating fish, but now I added the fish in regularly because of the omega- 3s and all that stuff.”* (P11).

Support from family members was also a key driver of fish/seafood consumption for some:

*“My mum … she was actually concerned that I couldn’t have seafood, but she was like ‘oh no I checked’ and was like ‘you can eat this’…I trust her because she has probably done her research.”* (P10).

Others who resided with relatives, and relied on them to do the household cooking, claimed that fish was often served as a family tradition:

*“…I live with my in-laws and they’re Catholic, so we have fish every Friday … I’m not a big seafood eater apart from fish and chips.”* (P8).

## Discussion

The primary aim of this study was to investigate the patterns of fish/seafood consumption among pregnant women, alongside an examination of their corresponding knowledge, attitudes, and behaviors pertaining to fish/seafood consumption throughout the antenatal period. Among pregnant women in our study, most knew that fish/seafood may contain the neurotoxin methylmercury and had received some advice to limit or avoid intake. Nevertheless, despite acknowledging the importance of good nutrition in pregnancy very few women could identify that fish/seafood contains important long chain omega 3 fatty acids or what benefits these important nutrients have for not only themselves but their growing baby as well. Likewise, a US qualitative study by Bloomingdale et al. in 2010 [10] employed focus groups to elicit the views of pregnant women (n = 22) regarding fish consumption during pregnancy. The authors found that most women knew that fish may contain methylmercury and had received advice to limit fish intake. Fewer women knew that fish/seafood contains DHA or what the function of DHA is. None of the women had received advice to eat fish [10].

It is well known that a growing fetus is high-risk for methylmercury exposure because of an increased susceptibility of the developing brain to this potential hazard. A recent population-based prospective cohort study used data from 5 European countries to assess the association of fish intake and methylmercury exposure during pregnancy with future metabolic syndrome in offspring [15]. The authors found that fish intake consistent with health recommendations (1 to 3 times per week) during pregnancy was associated with a 1-U decrease in metabolic syndrome score in children (β = −0.96; 95% CI, − 1.49 to − 0.42) compared with minimal fish consumption (< 1 time per week) after adjusting for maternal mercury levels and other covariates. No further benefit was observed with fish intake of more than 3 times per week [15].

Receipt of information to limit fish/seafood intake led some women in our study to eat less than they otherwise would have. Faced with a lack of available guidance, many avoided fish rather than possibly expose themselves or their babies to harm. Similarly, Greiner et al. [16] explored Canadian women’s views of healthy lifestyles during pregnancy. Participants described having difficulty interpreting and implementing nutritional guidelines especially around vitamin/mineral requirements and foods to steer clear of. Fish was a major source of confusion, with many women feeling unsure whether to avoid this foodstuff due to risks of methylmercury and food contamination or adding it into their diets for the long chain omega 3 fatty acid benefits [16]. Likewise, a qualitative study in New Zealand examined the discourses in nutrition information that pregnant women experience using 30 documents from three different platforms – media, government, and academia [17]. The authors identified the most confusing messages revolved around eating fish. The scrutinised documents contained information not only on the health benefits of consuming fish/seafood but the increased risk of methylmercury contamination that may occur when eating it. The documents also included recommendations to avoid eating fish varieties known to be susceptible to potentially high levels of methylmercury, to have a very limited consumption of other fish (identified to have lower levels of the toxin) and that the recommendations may change over time. The authors concluded that navigating such information requires a high level of literacy, comprehension, and education to follow and incorporate into everyday life [17].

In relation to overall dietary intake during pregnancy, women in our study focused heavily on things to avoid, particularly those with a potential food safety risk, rather than foods to include in their regular diet. We acknowledge that listeria exposure can result in serious consequences such as miscarriage or stillbirth. Nevertheless, cases of listeriosis in Australia are extremely rare (< approximately 0.02% of pregnancies per year) [18]. Women who choose to avoid fish/seafood due to possible bacterial contamination should be supported with information that enables them to identify and consume alternate dietary sources of EPA/DHA.

Regarding methylmercury toxicity, data collected by the Avon Longitudinal Study of Parents and Children measured this neurotoxin in maternal whole blood and umbilical cord tissue [19]. Offspring’s cognitive development was then monitored throughout childhood with no adverse associations noted. Interestingly, the authors reported beneficial associations with antenatal mercury levels for total intelligence quotient scientific reasoning, and birthweight in fish-consuming vs. non-fish consuming mothers [19]. These findings are like those observed in the Seychelles where fish consumption is very common and antenatal mercury levels, on average, ten times higher than US levels [9]. Despite concerns around methylmercury exposure for some participants in our study the nutritional benefits of fish/seafood were identified as an impetus for consumption.

Women in our study claimed that family support to eat fish/seafood during pregnancy was a highly motivating force. Likewise, Greiner et al. [16] described similar findings. However, none of our participants reported being given direct advice from their antenatal care provider to regularly consume fish/seafood as a way of increasing their EPA/DHA intake. In addition, very few participants had previously been advised by their GP to take a supplement if they avoided fish/seafood. To mitigate the lack of conversations around the benefits of eating fish/seafood in pregnancy, women reported accessing various online platforms for advice. In some cases, the nutrition information obtained was not locally applicable. Women also reported that the information they accessed was often conflicting and confusing. Similar qualitative research led by one of the lead authors of our study found that women were overly reliant on the internet while not having either the skill or capacity to critically evaluate the quality of nutrition information obtained [11]. Given the high volume of nutrition misinformation circulating the world wide web and social media, pregnant women, especially those with low food and health literacy, are at high risk of adopting inappropriate guidance. Previous qualitative research with antenatal care providers identified barriers to the provision of effective dietary counselling such as lack of education and limited resources to address important nutrition topics [20]. Of note is a recent review of the literature that reported the Australian accreditation standards for nursing and midwifery courses provide no content on nutrition [21]. Likewise, in the Royal Australian and New Zealand College of Obstetricians and Gynaecologists Integrated Training handbook, there is no specific module for nutrition [22]. A dietitian is the health professional best placed to advise pregnant woman regarding foods and food combinations to consume for optimum nutrition. Specifically, dietitians are food and nutrition experts who can provide individualised nutrition support to ensure pregnant women achieve omega 3 fatty acids targets by making specific alterations to their diet [23]. The need for a greater presence of dietitians in the antenatal care setting has been highlighted previously [24]. Ultimately, clinicians should place more energy into educating women about the neurocognitive benefits related to eating the recommended amounts of fish/seafood during pregnancy compared to negative associations as such information could be highly motivating.

In support of the Cochrane review previously referred to [3], current clinical guidance from the Royal Australian College of General Practitioners recommends that, if women do not consume enough fish/seafood per week to satisfy long chain omega 3 fatty acid requirements, a supplement with at least 500 mg DHA/day should be taken [25]. The supplement does not need to be more than 1000 mg DHA plus EPA overall, given higher doses do not appear to provide extra benefit. Furthermore, given some antenatal vitamin/mineral supplements do not contain long chain omega 3 fatty acids, pregnant women should be encouraged to seek out an appropriate omega 3 supplement to consume in addition to their antenatal vitamin/mineral supplement. With that said, a ‘food first’ approach involving regular consumption of fish/seafood instead of relying on omega 3 supplementation is most optimal. Despite research indicating that EPA and DHA levels increase in similar ways in the body when equal amounts of fish/seafood or fish oil are consumed [26], it is important to note that consumption of fish/seafood provides additional health-promoting nutrients including protein, vitamins A and D, iodine, and selenium [27] that are important throughout pregnancy. This is why the Australian Dietary Guidelines recommend a food first approach throughout pregnancy [28] and the lifespan, in general [29].

Pregnant women who experience sensory aversions to fish/seafood should be highly encouraged to consume an omega 3 supplement in place of eating fish/seafood, with vegetarian supplement options also available [30]. They should also be made aware of the differences between marine-based and plant-based sources of omega 3s, to support them in making an informed decision. For example, research indicates that supplementation with flaxseed oil does not lead to notable improvements in omega 3, including DHA levels in the blood [30, 31]. This is because flaxseeds are a rich source of the alpha-linoleic acid (ALA) version of omega 3, not the EPA and DHA versions of omega 3 that are abundant within fish/seafood [31, 32]. While ALA can be converted to EPA and DHA, this conversion process is highly inefficient in adults [31, 33]. For example, only approximately 8% of ALA is converted to EPA and 1% of ALA is converted to DHA in the body [34]. Additionally, the conversion rate of ALA largely depends on a well-planned diet encompassing adequate levels of energy, protein, and micronutrients including calcium, magnesium, zinc, copper, as well as vitamins B6 and B7 [35] which can be lacking in plant-based diets including vegetarian and vegan diets [36, 37]. Furthermore, omega 6 fatty acids such as Linoleic Acid, which is abundant in most ALA-rich oils [30], compete for the same desaturase enzymes required for conversion of ALA into EPA and DHA [38, 39]. Therefore, when high in the diet as they often are for those who are plant-based [40–42], omega 6 fatty acids can interfere with, and further reduce the conversion rate of ALA to EPA and DHA [38, 39]. When this takes place, ALA cannot provide health benefits similar to those provided by EPA and DHA [43]. This is why, ultimately, it is most optimal to obtain EPA and DHA directly from marine food sources [44] compared to plant-based sources, via a food first approach, where possible. For vegetarians and vegans, preliminary recommendations to sustain good EPA and DHA status include lowering omega 6 in the diet, especially if ALA serves as the primary source of omega 3, in addition to regularly consuming a preformed EPA and DHA supplement [30].

Currently, there is a paucity of research exploring the views and attitudes of women regarding fish/seafood consumption during the antenatal period in addition to the importance of EPA/DHA for a healthy pregnancy. Therefore, the findings of this study provide an important contribution to this gap in the literature. We acknowledge that this study is a small qualitative investigation, and that the views of pregnant women who participated in the interviews may not reflect those of pregnant women elsewhere. However, it was implemented according to best practice in qualitative research [45], and provides rich insight into the dietary knowledge, attitudes, and practices related to fish/seafood consumption among pregnant women living in the ACT. Further research exploring the fish/seafood knowledge, attitudes, and behaviours of pregnant women living in other Australian states is needed.

## Conclusions

This study provides good insight into pregnant women’s knowledge, attitudes, and behaviours regarding fish/seafood consumption throughout the antenatal period. Further research is needed to explore whether similar results are identified across all other Australian states and territories. To support adequate fish/seafood consumption among pregnant women, greater emphasis should be placed on the health benefits of regular fish/seafood consumption including the health promoting roles of omega 3 fatty acids for fetal growth and development. As food and nutrition experts, Dietitians are well placed to support pregnant women to meet fish/seafood recommendations. Additionally, those who cannot consume fish/seafood due to sensory aversions should be highly encouraged to consume a daily supplement that contains long chain omega 3 fatty acids.

## Data Availability

The data that supports this study are not publicly available in line with the ethics approval. Data are available from the corresponding author upon reasonable request.

## Abbreviations

FSANZ: Food Standards Australia and New Zealand
ACT: Australian Capital Territory
EPA: Eicosapentaenoic acid
DHA: Docosahexaenoic acid
US: United States
GP: General Practitioner
ALA: Alpha-linoleic acid
